# Cross-ECV consistency at global scale: LAI and FAPAR changes

**DOI:** 10.1016/j.rse.2021.112561

**Published:** 2021-09-15

**Authors:** Bernardo Mota, Nadine Gobron, Olivier Morgan, Fabrizio Cappucci, Christian Lanconelli, Monica Robustelli

**Affiliations:** a European Commission, Joint Research Centre, Via Enrico Fermi, 2749 21027 Ispra, VA, Italy.; b National Physical Laboratory, Earth Observation, Climate and Optical Group Hampton Rd. Teddington, Middlesex TW11 0LW, UK,

**Keywords:** LAI, FAPAR, Physical consistency

## Abstract

A framework is proposed for assessing the physical consistency between two terrestrial Essential Climate Variables (ECVs) products retrieved from Earth Observation at global scale. The methodology assessed the level of agreement between the temporal variations of Leaf Area Index (LAI) and Fraction of Absorbed Photosynthetically Active Radiation (FAPAR). The simultaneous changes were classified according to their sign, magnitude and level of confidence, whereby the respective products uncertainties were taken into consideration. A set of proposed agreement metrics were used to identify temporal and spatial biases of non-coherency, non-significance, sensitivity and the overall level of agreement of the temporal changes between two ECVs. We applied the methodology using the Joint Research Center (JRC) Two-stream Inversion Package (TIP) products at 1 km, those provided by the Copernicus Global Land Service (CGLS) based on the SPOT/VGT and Proba-V at 1 km, and the MODIS MCD15A3 at 500 m. In addition, the same analysis was applied with aggregated products at a larger scale over Southern Africa. We found that the CGLS LAI and FAPAR products lacked consistency in their spatial and temporal changes and were severely affected by trends. The MCD15A3 products were characterized by the highest number of non-coherent changes between the two ECVs but temporal inconsistencies were mainly located over the eastern hemisphere. The JRC-TIP products were highly consistent. The results showed the advantages of physically-based retrieval algorithms, in both JRC-TIP and MODIS products, and indicated also that, except for MODIS over forests, aggregated products using an uncertainty-based weighted average led to higher agreement between the ECVs changes.

## Highlights

• A new framework to evaluate physical consistency between LAI and FAPAR changes.
• Spatial and temporal agreements are assessed considering user confidence level.
• Products based on Radiative Transfer Model retrieval show best results.
• Results depend on both spatial scale and land cover types.

## 1 Introduction

Essential Climate Variables (ECVs) were defined in 2003 by the Global Climate Observing System (GCOS), and endorsed by the United Nations Framework Convention on Climate Change (UNFCCC) as “a physical, chemical, or biological variable, or a group of linked variables, that critically contribute to the characterization of Earth's climate” (GCOS, 2011, GCOS, 2016). ECVs derived from Earth Observation (EO) are now operationally delivered and they could be used in various policy and scientific domains emphasizing the need to provide full uncertainty budgets for them (Bojinski et al., 2014; Nightingale et al., 2019). Among terrestrial ECVs, the Leaf Area Index (LAI), defined as one half of the leaf area in the plant canopy within a given area, is one of the driving forcing parameters of net primary production, water and nutrient use, and carbon balance. FAPAR represents the fraction of photosynthetically active radiation absorbed by live/green vegetated elements (Sellers et al., 1997). They were used to monitor the state of the vegetation (Gobron et al., 2010; Polgar and Primack, 2011; Chen et al., 2019; Gobron, 2020), ecosystem productivity (Seixas et al., 2009) and to simulate a range of ecological responses to changes in climate and chemical composition of the atmosphere, including changes in the distribution of terrestrial plant communities across the globe in response to climate change (Wu et al., 2016). They are crucial to quantify the terrestrial sink sequestration on the global carbon budget (Cai et al., 2005).

Global dynamics of terrestrial processes can only be quantitatively and accurately assessed using long-term measurements over every region of the Earth. Since the early 2000s, optical sensors aboard satellites have been collecting spectral imagery from which LAI and FAPAR have been estimated. For example, data measured by Multi-angle Imaging Spectroradiometer (MISR), Moderate Resolution Imaging Spectroradiometer (MODIS), Advanced Very-High-Resolution Radiometer (AVHRR), Visible Infrared Imaging Radiometer Suite (VIIRS), PROBA-V, SPOT-VEGETATION, MEdium Resolution Imaging Spectrometer (MERIS) and more recently Sentinel-3 Ocean Land Colour Instrument were used to estimate globally LAI and FAPAR (Knyazikhin et al., 1998a, Knyazikhin et al., 1998b; Gobron et al., 1999, Gobron et al., 2019; Myneni et al., 2002; Pinty et al., 2011a, Pinty et al., 2011b; Baret et al., 2013; Zhu et al., 2013; Xiao et al., 2014; Claverie et al., 2016; Yan et al., 2018).

Comprehensive studies to validate these products, mostly under the framework of the Land Product Validation (LPV) subgroup of the Committee on Earth Observation Satellites (CEOS), have been conducted performing product inter-comparisons at global scale and at regional scale (Gobron et al., 2007; Weiss et al., 2007; McCallum et al., 2010; Ogutu et al., 2011; Fang et al., 2012, Fang et al., 2013; Camacho et al., 2013; Claverie et al., 2013; Martínez et al., 2013; Serbin et al., 2013; D'Odorico et al., 2014; Pickett-Heaps et al., 2014; Stern et al., 2014; Yan et al., 2016b; Zhang et al., 2020; Fuster et al., 2020; Bayat et al., 2020). These products were compared with several ground field measurements (Gobron et al., 2007; Camacho et al., 2013; Tao et al., 2015; Xiao et al., 2015; Gobron et al., 2019, Gobron et al., 2021; Brown et al., 2020; Fang et al., 2019; Fuster et al., 2020). Many global multi-temporal ECV land products based on the same, or combinations of, sensor imagery have been released and are now available as Climate Data Records (CDR). Considering that they are essential to characterise the earth system and to constrain land, biosphere or crops models, cross consistency of ECV products must be guaranteed. Recent efforts to identify the three-main cross-ECV inconsistency types (technical, retrieval and scientific level) were highlighted in Popp et al., 2020. The level of scientific soundness was crucial between ECVs that strongly interact, as do LAI and FAPAR. There was a strong need to check for physical consistency between land ECVs to ensure energy conservation when used in climate or land models (Sellers et al., 1997; Pinty et al., 2011a; Wang and Zender, 2010; Yuan et al., 2017).

LAI and FAPAR are physically linked by the radiation absorption theory as LAI, a state variable, represents the optical depth of the canopy in which radiation is absorbed by the leaves, *i.e.* the scattering elements (Ross, 1981). It is therefore expected that when LAI changes occur, FAPAR will also vary in the same direction (Myneni et al., 1989). The magnitudes of both changes must also be proportional. The exception to this rule occurs during the senescence period before the fall of autumn leaves, where FAPAR could decrease with a constant LAI (The single scattering albedo value of senescence leaves differ from the green ones (Houborg and Anderson, 2009)). This implies that the necessary condition for a significant FAPAR change to happen is a significant change in LAI. We proposed a framework to check for the physical changes consistency between these two ECVs taken into account their uncertainties. Fang et al. (2013) noted that some EO product uncertainties are only based on theoretical precisions that represent the algorithm's weakness and therefore are unable to fully represent the error propagation. Uncertainties cannot simply offer an indication as to whether the products meet the user requirements but are also key for the data assimilation within land surface models (Barbu et al., 2014; Boisier et al., 2014). It is within this context that the product uncertainties can offer an indication as to the significance of a temporal change and therefore be used to set a confidence level of any change.

Mota and Gobron (2017), Mota et al. (2019) proposed a framework that can be used for any combination of physically linked ECVs and we adapted the methodology to assess cross-ECV consistency between LAI and FAPAR.

In the present paper, we evaluated the level of agreement between LAI and FAPAR changes from three products: the MODIS products (Myneni et al., 2015), the Joint Research Centre Two-stream Inversion Package Products (Pinty et al., 2011a, Pinty et al., 2011b) based on MODIS Collection 6 surface albedo, and the Copernicus Global Land Service products based on SPOT-VEGETATION and PROBA-V data (Verger et al., 2014). We analysed the results for each product and assessed the spatial and temporal consistency, based on land cover types. We also explored the impact of spatial resolution.

In the next section, we detailed the three products and their retrieval algorithms, and described the methods used to evaluate their changes agreement. In Section 3, we summarized the results and in Section 4, we discussed and highlighted the main conclusions of the study.

## 2 Data

### 2.1 The Copernicus Global Land Service (CGLS) products

The CGLS LAI/FAPAR products were derived from top of canopy reflectances in visible, near infrared and shortwave infrared bands using Neural Network (NN) tools (Verger et al., 2014). The products were supplied every decadal (10 days) period at global scale with a spatial resolution of 1/112°. The dataset was based on SPOT-VEGETATION imagery and when the mission ended in May 2014, the retrieval algorithms were adapted to PROBA-V imagery by applying a spectral conversion on PROBA-V Top Of Canopy (TOC) reflectances to get SPOT/VGT-compatible ones, and rescaling the PROBA-V neural network (NN) outputs to SPOT/VGT ones (Baret et al., 2016). The processing of version 2 products also used a temporal smoothing and gap filling process based on the version-1 climatology to ensure temporal continuity and consistency (Verger et al., 2015). The NN algorithm capitalized on previous products (such as CYCLOPES Version 3.1 and MODIS Collection 5 ones) and was calibrated with VEGETATION reflectance values. In the CGLS products, the FAPAR values correspond to instantaneous values at 10:00, solar time. The uncertainties associated with these products were estimated using the root mean square values between the dekadal value and the valid daily estimates within the 10 days period excluding the climatological filled values. We used 20 years (January 1999 to December 2018) of valid data to ensure that only the higher quality observations were analysed (Smets et al., 2018). This means that each variable, *i.e.* LAI and FAPAR, has their own and separate retrieval algorithm. The potential physical link between the state variables were in the source of inputs data used in the NN, *per se*.

### 2.2 The MODIS products (MCD15)

The MODIS LAI/FPAR Collection 6 products, retrieved from data acquired from the combined TERRA and AQUA platforms (MCD15A2H), were generated every 8 days at 500 m spatial resolution over the globe (Myneni et al., 2015). The main algorithm used the daily surface reflectance data by inverting a 3-D radiative transfer model through a look-up table (LUT) (Knyazikhin et al., 1998a, Knyazikhin et al., 1998b). The method essentially searched the solutions in the LUT that best fit the observed bidirectional reflectance factors (BRFs) in the MODIS red and near-infrared bands based on predefined biome type, using MODIS land cover maps. Eight biome types were used as *a priori* information to constrain the vegetation optical and structural parameter spaces (Yan et al., 2016a). The outputs were the mean values of LAI and FAPAR averaged over all acceptable solutions, and the standard deviation served as uncertainty. The intrinsic physical link between LAI and FAPAR is therefore assured through the 3D-RT model computations. In addition, the products supplied a quality control mask indicating whether the values were derived from the main method or from a backup solution based on an empirical relation with Normalized Difference Vegetation Index (NDVI) for different biomes (Myneni and Williams, 1994; Knyazikhin et al., 1998a, Knyazikhin et al., 1998b). We used 16-years of data from January 2003 to December 2018. To ensure the best quality of observations, we excluded the pixels classified as affected by significant or mixed clouds and those derived by the back-up algorithm using the quality layer (https://lpdaac.usgs.gov/documents/624/MOD15_User_Guide_V6.pdf).

### 2.3 The Joint Research Centre Two-stream Inversion Ppackage products (JRC-TIP)

The JRC-TIP was developed at the Joint Research Centre (JRC) and the products were generated to bridge the gap between remote sensing products and large-scale global climate models (Pinty et al., 2011a, Pinty et al., 2011b). The products were based on an inversion algorithm of a two-stream model (Pinty et al., 2006) using the white-sky broadband albedo product in the visible and near-infrared domains derived from MODIS to infer the probability density functions (PDFs) of the model parameters of the vegetation layer, such the effective LAI, the effective single scattering albedo, the preferential forward or backward direction of scattering and the soil albedo. The data assimilation technique assumed constant prior values of these model parameters and their uncertainties. In addition, a snow mask was used to prior the background albedo (Pinty et al., 2008). These parameters were then utilized together, with their retrieved uncertainties, to estimate the PDFs of the broadband (visible and near-infrared) scattered (*i.e.* canopy albedo), absorbed by the vegetation layer (*i.e.* FAPAR) and transmitted through the vegetation layer and absorbed by the background. Uncertainties for LAI and FAPAR were the standard deviations relating to the diagonal of the posterior covariance matrix derived from prior PDFs, observations and model uncertainties. JRC-TIP products were derived from MODIS Collection 6 data every 16-days at 0.01° globally between 2002 and 2018. The input data were MCD43D59 (MCD43D60) for the white-sky albedo in the visible range, near-infrared) and the MCD43D31 products for quality information. Snow status quality layer (MCD43D40) was used for background prior information.

### 2.4 Global Land Cover map

Throughout this study, aggregation by land cover type was based on the ESA Climate Change Initiative (CCI) land cover product, epochs 2000, 2005 and 2015 (UCL-Geomatics, 2017). These global land cover maps were available at 300 m spatial resolution (http://maps.elie.ucl.ac.be/CCI/viewer (last accessed on 1/2/2019)). In our analysis, we adopted the global land cover class legend referred to CCI-LC and the appropriate aggregation into vegetation structure (see Table S1 in supplementary material), and used the different epochs to match the period under study (see Fig. S1 in supplementary material). Spatial conformity with each ECV product resolution was achieved by spatially aggregating the LC maps using a majority filter.

## 3 Method: Agreement framework

As both ECVs are physically linked, temporal consistency between these variables must be coherent, *i.e.,* a significant change in one should be reflected in the second. The agreement between changes in LAI and FAPAR products (∆) is expressed by the following conditions:

(1) ∆γ⟺Δμ

(2) ∆γ>0⟹Δμ>0or∆γ<0⟹Δμ<0

where ∆*γ* and ∆*μ* are LAI and FAPAR consecutive temporal changes, respectively. Whereas the first condition establishes the cause-and-effect - stating that for any change of LAI (∆*γ*) a change in the FAPAR value (∆*μ*) should occur - the second condition depicts the radiation absorption law – expressed by requirement that if LAI/FAPAR increases (decreases) then FAPAR/LAI itself increases (decreases), respectively.

**Fig. 1:**
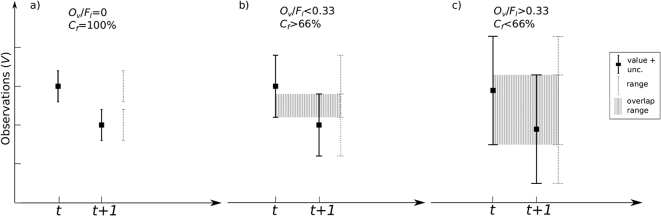
Change confidence level at a) 100%, b) greater than 66% and c) lower than 66%. Change confidence level is based on the overlap area (in grey) between two observations. Product uncertainties around the values represent the extent of possible observations and the ratio between the overlapping area and the total extent of two consecutive observations determines the confidence of change.

To analyse the level of agreement between the simultaneous LAI and FAPAR changes, we first quantified the frequency of the type of changes, *i.e.* negative and positive, that occurred in both variables according to conditions (1,2). In addition to the sign of the change, each change was assessed for its significance using the uncertainty to determine the level of confidence of the changes of one observation to the next. The confidence level, associated with each change allowed us to classify the changes into positive, negative or non-significant categories, was determined by the ratio between the overlapping region and the full range of values between two consecutive observations and their uncertainties. The full range is the interval of values that are possible between observations when considering their uncertainties to determine the maximum and minimum range, as depicted in Fig. 1. The overlapping range represents only the common values between the two intervals and expresses the level of similarity between the observations.

For example, large changes for which the associated ranges (value + uncertainty) do not overlap, indicate 100% of confidence in the change (see Fig. 1a). Changes where an overlap in the observations and their uncertainties was observed, highlight a degree of similarity and therefore a reduced level of confidence in the change (Fig. 1b and c). The proportion between the overlap and the full range of values provides the degree on how confident the change is: the confidence level. The greater the overlap, the lower the significance of the change. For example, if both observations have similar uncertainties but are small enough not to reach the following observation value, the overlap is below 0.33 resulting in a confidence level bigger than 66% (see Fig. 1b). When the observations have large uncertainties that overlap the change value, the resulting ratio is large, indicating a low confidence associated with the change.

The level of confidence is therefore given by:

(3) $${\left(C_{f}\right|}_{t}^{t+1}=100∙\left(1-\frac{O_{l}}{f_{l}}\right)$$

Where *O*_*l*_ is the change overlapping range, given by:

(4) $$O_{l}=\left\{\begin{array}{c} \left(v_{t+1}+u_{t+1}\right)-\left(v_{t}+u_{t}\right),v_{t+1}<v_{t}\\ \left(v_{t}+u_{t}\right)-\left(v_{t+1}-u_{t+1}\right),v_{t+1}>v_{t} \end{array}\right)$$

and *f*_*l*_ the full range is given by:

(5) $$f_{l}=\left\{\begin{array}{c} \left(v_{t}+u_{t}\right)-\left(v_{t+1}-u_{t+1}\right),v_{t+1}<v_{t}\\ \left(v_{t+1}+u_{t+1}\right)-\left(v_{t}-u_{t}\right),v_{t+1}>v_{t} \end{array}\right)$$

where *v* is either LAI or FAPAR and *u* is the associated uncertainty for consecutive observations at *t* and *t* *+* *1.*

All changes for LAI and FAPAR were analysed and classified according to the type of changes that occurred simultaneously. The three possible change classifications were: 1) an increase, 2) a decrease or 3) no confidence in the change. A change, to be considered as increase or decrease, must have at least a C_f_ value above 50%. Elsewhere, it was classified as a non-significance change (NC). In total, when considering the ECV simultaneous changes, nine possible classification combinations can occur.

**Fig. 2:**
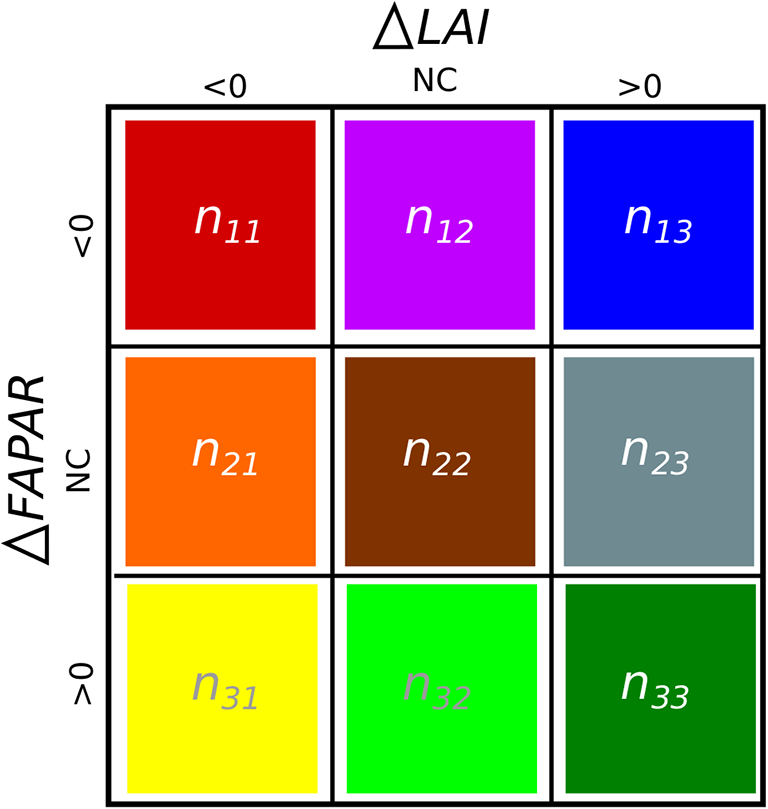
Contingency table representing the LAI and FAPAR change classifications based on the significance of change observed simultaneously within each product. Diagonal elements represent coherent cases of increase (n33), decrease (n11) events and no-confidence changes in both products (n22).

To evaluate the agreement between product changes, all nine possible types of intra-product changes were counted and used to populate the contingency table as framed in Fig. 2. This contingency table, also known as a confusion matrix, was used to derive the agreement and bias metrics. Physically coherent situations can assume three forms:

● Significant increase in LAI leading to a significant increase in FAPAR, depicted in *n*_*33*_,
● the inverse significant change leading to a decrease depicted in *n*_*11*_ and,
● the cases for which both changes in LAI and FAPAR are simultaneously classified by a low confidence level and therefore considered non-significant, depicted in *n*_*22*_.

Physical non-coherence was represented by the contradicted cases where significant LAI increase/decrease led to significant FAPAR decrease/increase, represented by *n*_*13*_/*n*_*31*_, respectively.

The agreement between LAI and FAPAR changes was based on metrics developed for classification accuracy assessment (Story and Congalton, 1986). Overall accuracy, renamed hereafter as overall agreement (*OA*), is determined by the total percentage of coherent simultaneous changes, represented by the diagonal elements of the confusion matrix (Fig. 2), given by:

(6) $$\mathrm{OA}=\frac{\sum_{i=1}^{3}n_{\mathrm{ii}}}{N}$$

where *n*_*ii*_ is the number of cases that fell into each coherent change classification type (*i* = 1, 2, 3) and *N* is the total number of considered cases.

Producer and user accuracy, also known as omission and commission errors (Janssen and Vanderwel, 1994), are combined into a single metric known as dice coefficient (Dice, 1945; Forbes, 1995). This coefficient has a probabilistic interpretation that if a given LAI product identified a change, the dice coefficient (DC) is equal to the conditional probability that the FAPAR product will also identify the corresponding coherent event (Fleiss et al., 2003). The dice coefficient is hereafter referred to as an increase sensitivity (*S*_*i*_) for the growing season, or a decrease sensitivity (*S*_*d*_) for senescence period. These are respectively given by Eqs. (7), (8):

(7) $$S_{i}=\frac{2∙n_{33}}{2∙n_{33}+n_{32}+n_{31}+n_{23}+n_{13}}$$

(8) $$S_{d}=\frac{2∙n_{11}}{2∙n_{11}+n_{12}+n_{13}+n_{21}+n_{31}}$$

The non-coherent bias (*B*_*nc*_) provides the information on the degree of balance between the two types of significant non-coherent cases. This bias refers to the percentage of all cases of the difference between the significant changes where LAI and FAPAR vary incoherently (Eq. (9)).

(9) $$B_{\mathrm{nc}}=\frac{n_{13}-n_{31}}{N}$$

The non-matching non-significant bias (*B*_*ns*_) evaluates the degree of balance between both non-significant changes between the ECVs. It is given by the percentage of all cases of the number of non-matching non-significant LAI changes and the ones associated with FAPAR (Eq. (10)).

(10) $$B_{\mathrm{ns}}=\frac{\left(n_{21}-n_{23}\right)-\left(n_{12}-n_{32}\right)}{N}$$

### 3.1 Temporal and spatial consistency evaluation

We determined the level of changes agreement of the three EO products, applying the framework described in the previous section. The level of agreement was evaluated for its spatial and temporal consistency based on the following approaches:

● Temporal consistency was assessed using a map approach where, for each temporal change, the simultaneous changes, for specific continent and land cover type were classified and used to determine the level of agreement and biases. The integration with respect to time of the agreement and bias metrics results in a time-series from which one can evaluate the temporal consistency. Short-term consistency was assessed using the monthly climatological averages of the agreement metrics and the long-term consistency was evaluated by testing for a monotonic trend in the agreement metrics, using the Seasonal Mann-Kendall test (Hirsch et al., 1982, Hirsch and Slack, 1984)
● Spatial consistency was evaluated using a pixel based approach where the time-series of simultaneous classification changes, throughout the full period that occurred for each pixel, were used to determine the level of agreement, resulting in a map of agreement and bias metrics. These maps were used to estimate for spatial consistency within and between each land cover type by continent. The consistency was calculated by testing for mean and distribution differences between the products using the Welsh two-way (Welch, 1947) and the Kolmorov-Smirnov two-way tests.

### 3.2 Agreement sensitivity to the change confidence level

As the confidence level associated with each temporal change is a key parameter to determine its significance, we evaluated the impact of different *C*_*f*_ thresholds on agreement between the ECV's changes. We first looked at the overall agreement (OA) results by varying the *Cf* threshold value (25%, 50% and 75%) but by applying the same values to both LAI and FAPAR changes. This was done at global scale for the entire period of the datasets. We then focused on a particular year and area, and evaluated the impact on non-significant bias between the ECVs, using different *C*_*f*_ thresholds (at 1% scale steps) applied independently to each ECV. This analysis indirectly allowed us to examine how, and if, the relation between LAI and FAPAR uncertainties varies within each product. We restricted our analysis over Southern Africa, characterized by the presence of the four dominant land cover classes representing the main vegetation structures -croplands, forests, shrubs, grasslands- and during the 2012 representing an average season, selected randomly from the middle period of the ECV datasets.

### 3.3 Spatial resolution impact on the ECV agreement

To assess the spatial resolution impacts on the level of agreement, the framework was applied at four coarser resolutions (0.05°, 0.10°, 0.25° and 0.50°) for 2012 over Southern Africa [0–35°S; 8–43°W]. The method for aggregating the products to a coarser resolution is based on a weighted average (*R*_*s*_) approach, where the weights are calculated based on the associated product uncertainties.

The weighted average for each grid-cell was given by Eq. (11)

(11) $$R_{s}=\frac{\sum w_{i}\alpha_{i}}{w_{i}},w_{i}=\frac{1}{\delta_{i}^{2}}$$

where *δ*_*i*_ were the uncertainties associated to each ECV value *α*_*i*_ within each grid cell *i*^*th*^*,* and *w*_*i*_ the associated weights.

Ultimately, the uncertainty associated to Rs, *σ*_*s*_ is given by Eq. 12.

(13) $$\sigma_{s}=\sqrt{\frac{1}{\sum w_{i}^{2}}}$$

## 4 Results

### 4.1 Confidence threshold impact

**Fig. 3:**
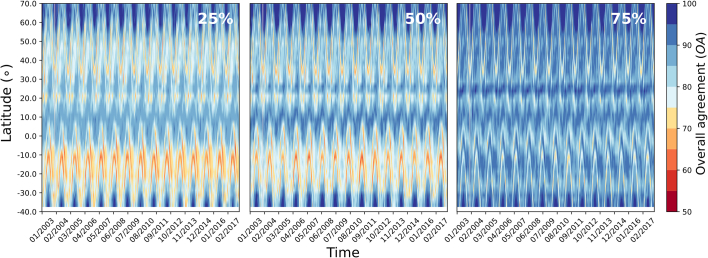
Hovmöller diagram of the overall agreement between the MCD15 LAI and FAPAR products using a) 25%, b) 50% and c) 75% confidence threshold of change between 2001 and 2018 at 0.5° spatial resolution.

The level of agreement between the products' changes was dependent on the chosen confidence threshold *C*_*f*_ associated with the ECV temporal changes. Choosing a smaller or larger threshold may result in different strengths of agreement. This was highlighted in Fig. 3 displaying the overall agreement zonal average (*OAz*) values when applying a 25%, 50% and 75% confidence threshold for the MODIS products aggregated at 0.5° spatial resolution.

The figure showed that on average a higher overall agreement was obtained by applying a higher threshold. This meant that by setting a higher threshold requirement (*i.e.* requiring a larger change significance), higher *OAz* were obtained. The same effect was observed for the other products (not shown here).

**Fig. 4:**
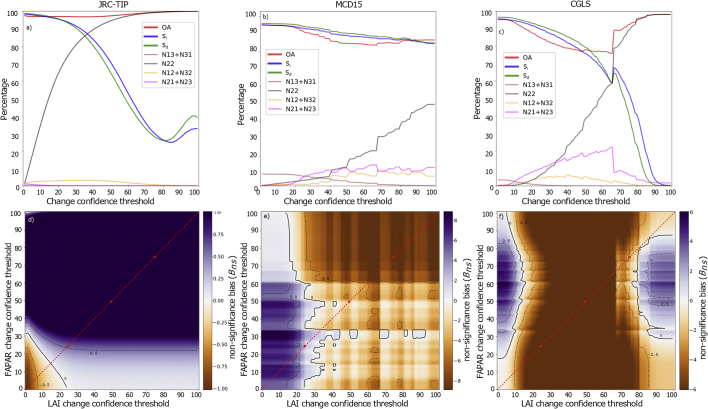
Change classification and overall agreement variation with confidence threshold (upper panels) and non-coherent bias (bottom panels), between the LAI and FAPAR products changes (JRC-TIP, MCD15 and CGLS on the left, middle and right panels, respectively) for the tropical forests over Southern Africa for 2012. Top panels variation was represented by the red line in the bottom panels. (For interpretation of the references to colour in this figure legend, the reader is referred to the web version of this article.)

However, as shown in Fig. 4, this relation was non-linear and dependent on land cover.

Depending on the land cover type, each dataset follows a specific dynamic between agreement and confidence threshold. Fig. 4 illustrates how the agreement metrics vary with the confidence threshold, for each dataset over forests in southern Africa during 2012. The upper panels related to the variation in agreement metrics that resulted from applying equal confidence thresholds for individual ECV, and the lower panels to the non-significant bias (*B*_*ns*_) when choosing independent confidence thresholds for each ECV. In the bottom panels, the red line indicated the same threshold level applied to the LAI and FAPAR changes that resulted in the upper panel metrics.

Matching non-significant changes always increased with confidence threshold (grey line in top panels). Its rate of increase varied with product: it was higher for the JRC-TIP (Fig. 4a) and lower for the MCD15 (Fig. 4b). This was due to the JRC-TIP's larger uncertainties values relative to the ECV absolute values. Indeed, the prior value (uncertainty) for LAI was set to 1.5 (5). For both MCD15 and CGLS, relative uncertainties were smaller compared to the JRC-TIP ones, as they do not correspond to actual standard deviations of PDFs, and therefore led to more changes being classified as significant. However, as non-significant changes contributed positively to OA if they were simultaneous, these contributed positively to *OA*. In case of the sensitivities to increase (*S*_*i*_) and decrease (*S*_*d*_), the growth in the number of matching non-significance can have a high impact due to the reduction of coherent changes classified as significant (*n*_*11*_ or *n*_*33*_, in Fig. 2).

The example also showed a decrease in the number of non-coherent changes (*n*_*13*_ + *n*_*31*_) for larger confidence thresholds (brown line top panels). Although this was common to all products, for the JRC-TIP product non-coherence cases were not observed for this case, whereas the CGLS and MCD15 required a *C*_*f*_ threshold of 25% and 75%, respectively, to result in less than 1% of changes being classified as non-coherent (brown line top panels). This showed that non-coherence was more frequent in the MCD15 than in the CGLS or the JRC-TIP product, indicating that the lower relative uncertainties allowed for non-coherent changes to be classified as significant.

In terms of non-matching and non-significant changes, *e.g.* the lack of balance in the confidence of changes between the ECVs, the JRC-TIP product shows consistency by having a higher number associated with LAI (*n*_*21*_ *+* *n*_*23*_) than FAPAR (*n*_*12*_ *+* *n*_*32*_) throughout all *C*_*f*_ thresholds (magenta and yellow lines, left upper panel). The exception (n12 + n32 > n21 + n23) is only at very low *Cf* that upon inspection showed that these are associated with very low LAI and FAPAR values. For the MCD15, the opposite is observed. More changes in FAPAR than in LAI are classified as non-significant above the *C*_*f*_ thresholds of 40% (middle upper panel). As for the CGLS, the results show that independently of the chosen *C*_*f*_, non-significant changes are always higher for FAPAR than for LAI (right upper panel).

Choosing independent *C*_*f*_ thresholds for each ECV led to different *B*_*ns*_ and therefore allowed us to identify the kind of relation between the ECV's uncertainties. Fig. 4 showed the *B*_*ns*_ distribution for all possible *C*_*f*_ for the three products. A negative bias for the JRC-TIP product, meaning more non-significant FAPAR changes than LAI ones, only occurred when considering low *C*_*f*_ for both LAI and FAPAR. In the case of the MCD15 product, this only occurred for the higher *C*_*f*_, mainly associated with FAPAR changes (Fig. 4e). For the CGLS, a negative bias dominated all *C*_*f*_ between 20 and 65% associated with LAI (Fig. 4f). These results reflected the type of relation between each ECV uncertainty. For the JRC-TIP product, in relative terms, uncertainties tended to be always lower for FAPAR than for LAI. This was a direct consequence of how error propagated within the two-stream model characterized by a negative power exponential between FAPAR and LAI. In the case of the MCD15 product, the results showed a contrasting relation, where FAPAR uncertainties tended to be higher, in relative terms, than the LAI ones. In addition, the MCD15 products were characterized by the biggest bias values among the three products and by a lower number of precision units leading to the unsmooth patterns shown in the *B*_*ns*_ distribution. In the case of the CGLS, positive bias only occurred for the lower or higher end of *C*_*f*_ thresholds associated with the LAI changes, indicating a dichotomous relation between the ECV's uncertainties.

The best compromise between LAI and FAPAR changes, the one that showed balance between the number of significant cases occurring simultaneously in both ECVs, was the one that led to zero non-significant bias. For this forest case, this can only happen when a *C*_*f*_ around 16%, 33% and 80% is applied to the JRC-TIP, MCD15 and CGLS datasets, respectively. For other land cover types, these values changed (not shown), and could be susceptible to change of study area and time period. Although one can find the *C*_*f*_ value that optimized all possible cases (spatially and temporally), it would prevent a proper consistency analysis study. Here, the objective was to analyse the product's consistencies under equal terms. Therefore, it was for this reason that we chose a *C*_*f*_ of 50%, to be applied throughout the rest of study, representing the standard statistical threshold for decision making.

### 4.2 Spatial consistency

**Fig. 5:**
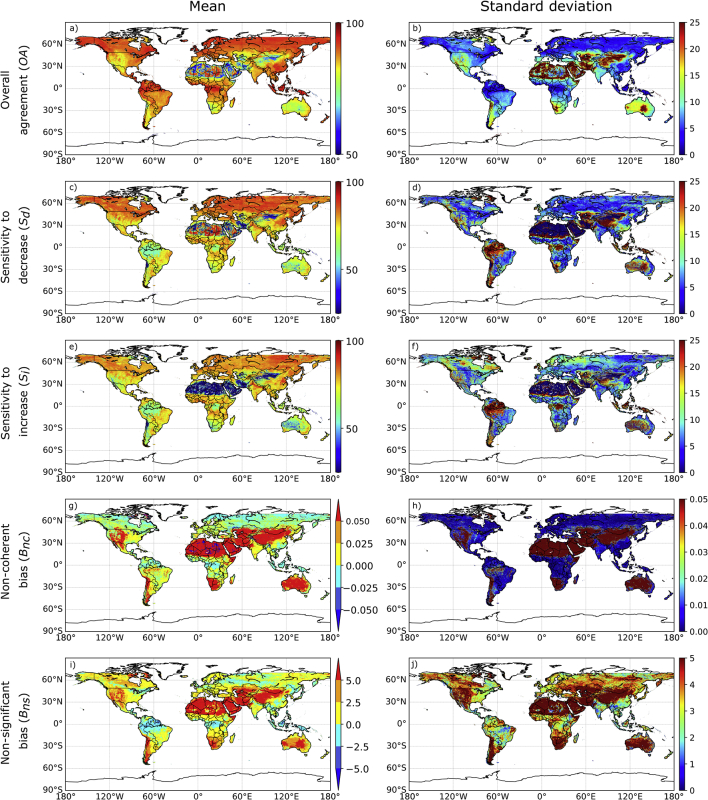
Spatial distribution of the average of the three products agreement metrics for (left panels) and the associated standard deviation (right panels) using the 50% confidence threshold of change.

The level of agreement between the ECVs changes was determined using the 50% confidence threshold, and its spatial consistency was evaluated for each product. To highlight the differences between the product's results, we plotted the mean of their metrics and the associated standard deviations in Fig. 5**.**

In terms of overall agreement *(OA) (*Fig. 5a*)*, large spatial variations were found with high values associated with areas where vegetation is more active and low values over deserts and sparsely vegetated areas. The same pattern can also be observed for both sensitivity metrics (Fig. 5d and e), except over tropical forests characterized by sensitivities as low as the ones over sparsely vegetated areas. Over these areas, ECV changes were relatively small or constant compared to their uncertainties in the three products. This resulted in a larger number of non-significant cases leading to lower sensitivities. The results also showed that, on average the products exhibited higher sensitivity to decrease *(S*_*d*_*)*than to increase *(S*_*i*_*)*.

As indicated by the larger standard deviation values (right panels), discrepancies between the products are also observed. These, in terms of *OA,* were larger over deserts and sparsely vegetated areas (Fig. 5b). In terms of sensitivities, discrepancies were observed over tropical forests and sparsely vegetation for both sensitivities (Fig. 5d and e), and over Boreal forests for only *S*_*i*_ (Fig. 5e).

In terms of error metrics, non-coherence bias (*B*_*nc*_) is positive over agricultural and sparsely vegetated areas and negative over forests (Fig. 5g). Except for deserts and sparsely vegetated areas - where the difference is higher - mean non-coherence bias and associated standard deviation is small indicating consistency between the products (Fig. 5h). In terms of non-significant bias (*B*_*ns*_), a similar spatial pattern to *B*_*nc*_ was observed but characterized by higher values and, as the standard deviation values shown, by larger intra-product differences (Fig. 5j).

**Fig. 6:**
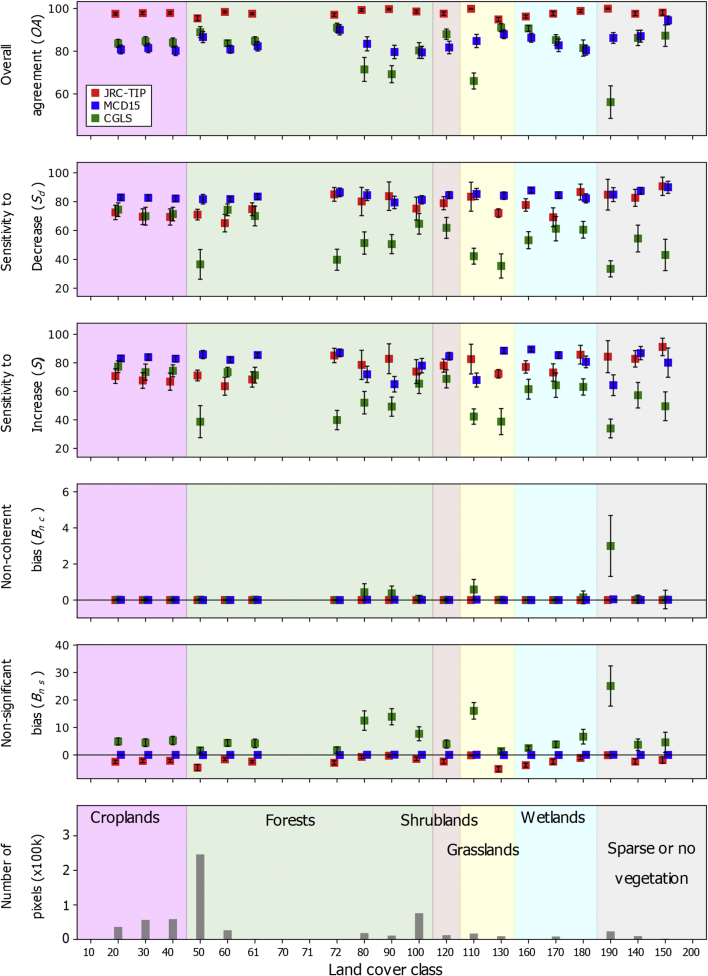
Spatial mean agreement metrics between the three LAI and FAPAR datasets by land cover class over the South American continent using the 50% confidence threshold of change (error bar indicates one standard deviation).

We also summarized the results by land cover class over South America (Fig. 6). Additional plots for other continents are available in the Supplement Document. On average, low agreements and error biases were normally restricted to the land cover classes with lower vegetation content, namely shrubs and grasslands. JRC-TIP products were always characterized by the highest agreement, while the CGLS were generally associated by the lowest agreement between the ECVs changes (Fig. 6a). In terms of sensitivity to decrease, *S*_*d*_, the MCD15 products on average provided higher values over croplands and most forest types (Fig. 6b). Exceptions were only observed over Australia where *S*_*d*_ for the JRC-TIP was more sensitive to decrease (Fig. S3). CGLS products had the lowest sensitivity, with only a few exceptions, over forests. Over shrubs, the JRC-TIP products had the highest one over Asia, Australia and Africa whereas it was the MCD15 for the remaining continents. Over the two grassland types, these same products can show alternating sensitivities, depending on the continent. Regarding the sensitivity to increase, *S*_*i*_, the results were identical to *S*_*d*_ but characterized by slightly lower values. In terms of error bias, the CGLS and JRC-TIP products were characterized by positive and negative non-significant bias, respectively (Fig. 6e). In contrast, a very small positive bias was associated with the MCD15 products. Regarding non-coherency, only the CGLS dataset showed positive bias over a restrictive set of LCs, such as grasslands over the Southern American continent (Fig. 6d). Intra-class spatial consistency, illustrated by the error bars associated with *B*_*ns*_ variability, tended to be higher for grasslands and shrubs independently of the product and continent. On average, the MCD15 products were characterized by a lower variability and the CGLS by the highest. This meant that the CGLS was characterized by a degree of non-coherency spatial heterogeneity.

### 4.3 Temporal consistency

#### 4.3.1 Global pattern

**Fig. 7:**
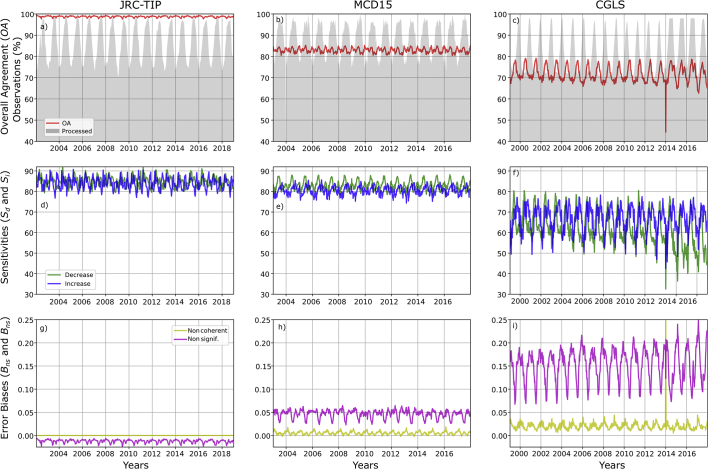
Global temporal profiles of the change agreement metrics (row panels) - Overall Agreement (red), sensitivity to increase (blue) and decrease (green), non-significant (magenta) and non-coherent (yellow) biases - between the simultaneous changes of the LAI and FAPAR products (TIP, MCD15 and CGLS on the left, middle and right panels, respectively), using the 50% confidence threshold of change. (For interpretation of the references to colour in this figure legend, the reader is referred to the web version of this article.)

The temporal consistency approach illustrated that the products were characterized by considerable differences in terms of magnitude and trend, of the agreement and error bias metrics. The results at the global scale for the JRC-TIP (left panels), the MCD15 (middle panels) and the CGLS product (right panels) are displayed for the three agreements and the two-bias metrics in Fig. 7. For the overall agreement (*OA*)*,* the JRC-TIP products had the highest values with a mean value for the full period of 98.8% whereas we found 83.1% for MCD15 and 71.0% for CGLS.

There was no significant difference between both sensitivity metrics (84.8% and 83.9%) for the JRC-TIP product. In the case of the MCD15, the *S*_*d*_ (83.0%) was slightly greater than the *S*_*i*_ (80.5%). CGLS products was not only characterized by lower mean values (61.0% and 66.7% for *S*_*d*_ and *S*_*i*_, respectively) but a clear negative trend was detected for *S*_*d*_, with a drop post mid-2004 (corresponding to the VGT-1 and VGT-2 transition) and even a greater drop for the post-2014 period (transition from VGT to PROBA-V).

Non-coherent bias, *e.g. B*_*nc*_, was not detected for the JRC-TIP products and the MCD15 had a small positive mean bias of 0.05%. For the CGLS, *B*_*nc*_ variations were not only bigger but their mean magnitude was of 0.15%. One can notice also a large peak during the 10-day transition period of 2014. The three products had contrasting non-significant bias (B_*ns*_) results. Whereas the JRC-TIP dataset is characterized by a low mean negative bias (−0.01%), the MCD15 has a mean positive bias (0.05%). The CGLS was not only characterized by a much larger mean positive bias (0.15%) but also by a larger seasonal variation following a positive trend.

One can also notice that the percentage of processed pixels relative to the number of available land pixels (grey shaded areas, upper panels) was characterized by the expected seasonal oscillation. The mean value over the full period was similar for JRC-TIP and MCD15 (58.7% and 62.6%, respectively). As for the CGLS, this percentage was smaller, reaching mean values below 50% or 85% (pre-or post-2014 period), associated with the sensor transition from VGT to PROBA-V. In summary, the agreement and bias metrics for the CGLS product were characterized by a larger temporal and seasonal variability. Not only was the magnitude of values larger but also the seasonally based oscillation clearer. Due to the smaller and noisier oscillations, the seasonal pattern was not as apparent for the JRC-TIP and MCD15 products.

#### 4.3.2 Seasonality – Short term consistency

**Fig. 8:**
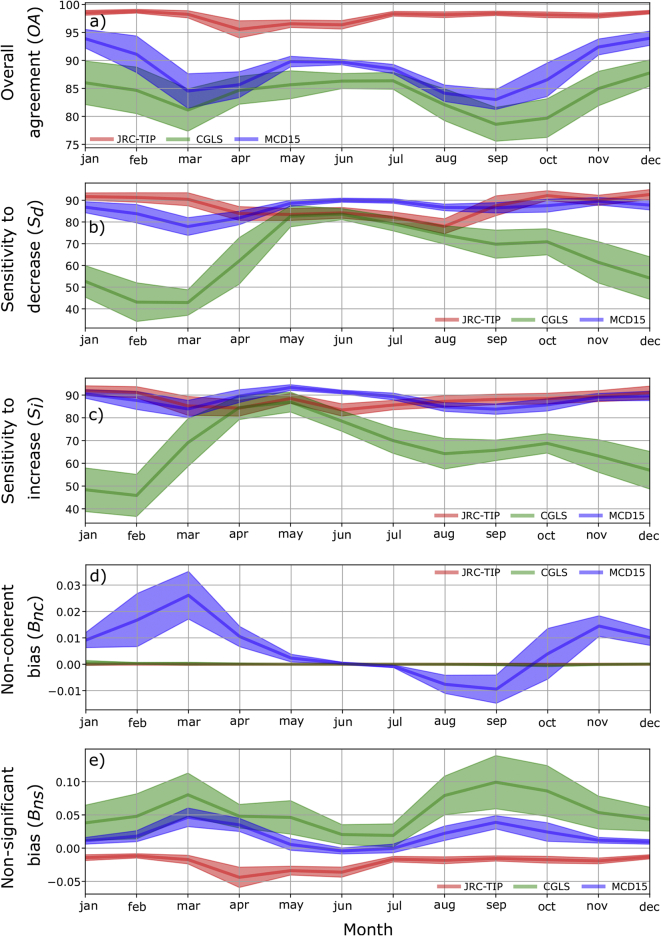
Mean seasonal profiles for the change a) overall agreement, b) sensitivity to decrease, c) sensibility to increase, d) non-coherent and e) non-significant biases between the LAI and FAPAR changes products over the North American continent using the 50% confidence threshold of change (shaded area represents one standard deviation).

Fig. 8 illustrated the average annual variation of the agreement metrics over North America for the three products. These represented the climatological seasonal variation and, for this continent, with only one clear season occurring during the year. For the JRC-TIP, *OA* and *S*_*i*_ variations were minimal and a larger variation was only observed with *S*_*d*_ (Fig. 8b). Non-significant bias was always negative with larger values occurring from April to June (Fig. 8e). As for the MCD15, the annual *S*_*d*_ and *S*_*i*_ variations were slightly larger than for the JRC-TIP between the May–August (Fig. 8b) and April–June (Fig. 8c) period, respectively. Non-coherence bias for the MCD15 tended to be always positive, meaning significant LAI increase with simultaneous FAPAR decrease. The exception occurred during August and September (small negative bias) meaning that during this period non-coherence was characterized by significant FAPAR increase and LAI decrease.

The CGLS followed a clearer seasonal variation characterized with lower *OA*, *S*_*d*_ and *S*_*i*_ in comparison to the other products. In this region, the winter months were always characterized by the lowest agreements (*OA*, *S*_*i*_ and *S*_*d*_). In summary, CGLS exhibited a clear seasonal pattern, meaning that the lack of agreement between the ECV's change was highly dependent on the season and therefore, lacked short term temporal consistency. The MCD15 products' change agreement was not as affected by seasonality but non-coherence bias can be an issue. Seasonal variations over the other continents (not shown) showed similar results.

#### 4.3.3 Temporal trends – Long term variation

**Fig. 9:**
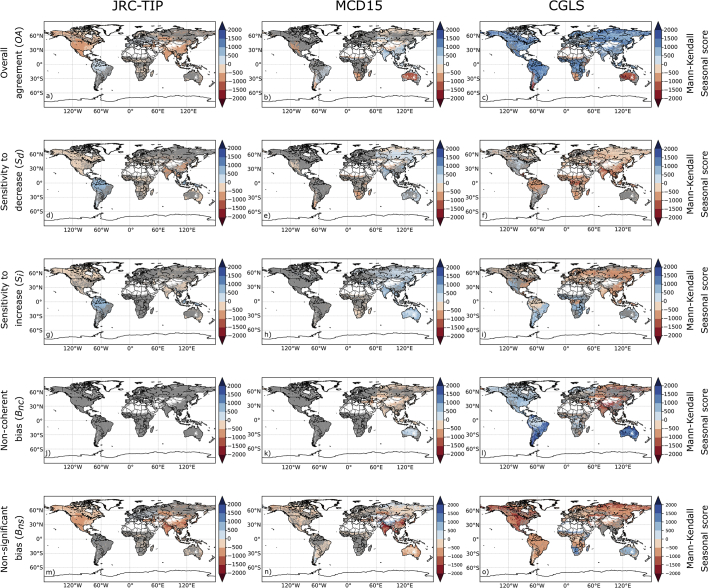
Spatial distribution of the Seasonal Mann-Kendal trend scores for the overall agreement for a) JRC-TIP, b) MCD15 and c) CGLS LAI and FAPAR products using the 50% confidence threshold of change. Grey indicates areas for which no significant monotonic seasonal trend was detected.

The trends of both agreement and error bias time-series were analysed at global scale. Fig. 9 shows the spatial distribution of Mann-Kendall seasonal score over the areas where it was significant for each metric using the JRC-TIP (left panels), MCD15 (middle panels) and CGLS (right panels) dataset.

JRC-TIP depicted significant negative trends for *OA* and for sensitivities over North America and the south Asia region. In contrast, positive trends were observed in South America. Trends for *B*_*ns*_ were always negative and mainly over areas for which *OA* also had a negative trend. The JRC-TIP dataset did not show any *B*_*nc*_ trend.

For the MCD15, significant *OA* trends were mainly detected over the eastern hemisphere: negative in Southern Africa and Australia and positive in Asia. The same trends as *OA* were detected in *S*_*i*_ and *S*_*d*_ values, except for a sign change over Australia. Only the regions over south Asia and Australia showed trends of opposite sign for *B*_*nc*_. As for *B*_*ns*_, the trends were mainly negative with significant high scores located in India and China, that can be associated with irrigated croplands.

The CGLS dataset was characterized by the highest geographical extent over which a significant trend was detected across all metrics. For example, trends of *OA* with significant high positive scores were found over most areas covered by vegetation. One exception was the negative trends located over sparsely vegetated areas in southern Africa and Australia. Contrasting trends for *S*_*i*_ and *S*_*d*_ occurred over non-forested vegetation in Africa. Over the western hemisphere and Australia *B*_*nc*_ were characterized by a positive bias trend whereas a negative trend was detected over Asia. As for the *B*_*ns*_, much of the areas were characterized by negative trends except for sparsely vegetated areas over Australia and southern Africa.

**Fig. 10:**
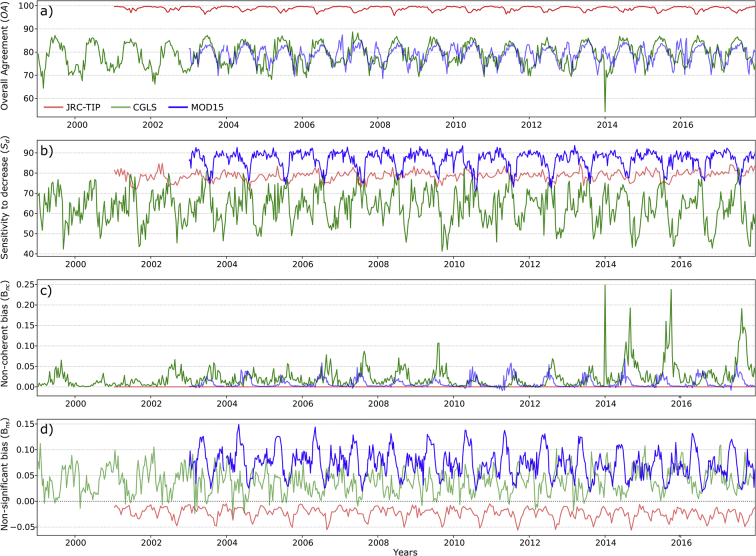
Time-series for a) overall agreement over croplands in North America, b) Sensibility to decrease over tropical forest in South America, c) Non-coherence bias over sparsely vegetated areas in Australia, and d) Non-significant bias over croplands over India for the JRC-TIP (red), CGLS (green) and MCD15 (blue). (For interpretation of the references to colour in this figure legend, the reader is referred to the web version of this article.)

In summary, the results demonstrated that datasets had significant trends in the agreement metrics over different areas, and that these were of the same or opposing sign depending on the product. As one example, the results over croplands in North America are plotted in Fig. 10. Whereas a small but significant negative OA trend was detected for the JRC-TIP, a positive trend was detected for CGLS (Fig. 10a). The JRC-TIP negative *OA* trend was associated with a negative *B*_*ns*_ trend, meaning that over time more FAPAR changes were classified as non-significant in comparison with the LAI ones. This caused lower number of simultaneous changes being classified as non-significant or coherent, thus contributing less to the overall agreement. In contrast, the CGLS product was categorised by a negative *B*_*ns*_ trend of positive bias values, meaning that there were less non-significant LAI changes over time in comparison with the FAPAR ones. The second example, over tropical forest in South America, also demonstrated opposite trends associated with *S*_*d*_, where the JRC-TIP/CGLS products were characterized by a positive/negative trend, respectively (Fig. 10b). The third example showed that trends of *B*_*nc*_ were mostly found with the CGLS dataset: over sparsely vegetated areas in Australia. In this case, not only was the bias value higher compared to the other products, but a significant positive trend was detected (Fig. 10c). This meant that the number of changes where LAI decreases and FAPAR increases compared to the number of changes where LAI increases and FAPAR decreases was growing in time. The main contribution to the trend was the transition between the pre-and post-2014 period, meaning that the PROBA-V based products were more prone to non-coherence.

The three products also manifested aligned trends over similar areas. For example, the *B*_*ns*_ negative trend over croplands in India for both JRC-TIP and MCD15 (Fig. 10d). Here, the trends meant that the balance between the number of non-significant simultaneous LAI and FAPAR changes was varying over time. But whereas for the JRC-TIP this difference was becoming more negative, meaning more bias, for the MCD15 the difference was becoming less positive, and therefore less biased.

The outcomes also suggested that trends were not dependent on the type of vegetation (*e.g.* the land cover classes), as the trend results for the same land cover can differ between continents. One example was over the tropical forests for the JRC-TIP datasets, where only South America was characterized by significant agreement trends. Another example of land cover independence was for the MCD15 product, where the significant trends were mostly restricted to the eastern hemisphere.

### 4.4 Spatial resolution impact

**Fig. 11:**
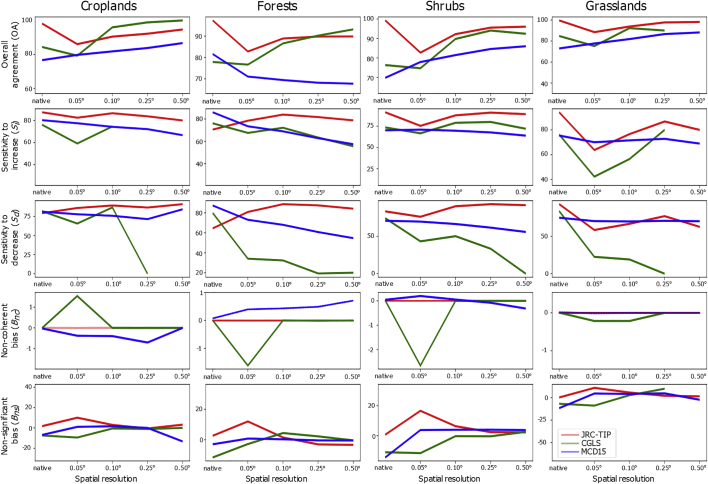
Agreement metrics variation by spatial resolution between the simultaneous changes of the JRC-TIP (red), CGLS (green) and MCD15 (blue) LAI and FAPAR for agriculture (left column panel), forest (second column panel), shrub (third column panel) and grasslands (right column panel) classes over Southern Africa [0–35°S; 8–43°W] using the 50% confidence threshold of change for year 2012. (For interpretation of the references to colour in this figure legend, the reader is referred to the web version of this article.)

Our framework was applied at various spatial resolutions to evaluate the scale impacts at the level of agreement between the ECV simultaneous changes. Fig. 11 showed the variation in agreement and bias errors between the three datasets over the main four land cover classes over the southern African region during 2012. Except for the MCD15 over forests, on average, the highest overall agreement is either observed at the native resolution or at the lowest possible resolution (upper panels). This means that the higher the number of pixels used in the aggregation, the lower the chance of both ECVs not agreeing. However, except for the JRC-TIP products or over grasslands, on average both sensitivities (Si and Sd) for the MCD15 and CGLS products decrease with resolution, indicating a loss of sensibility to detect a significant change between the ECVs, especially during the senescence period (Sd). For the MCD15 products over forests, both OA and sensitivities decrease, and Bnc increases, for lower resolutions. This means that the associated product uncertainty to weight the aggregated average leads to worse results.

## 5 Discussion and conclusions

In this paper, we evaluated the spatial and temporal consistency between LAI and FAPAR changes for three operational EO datasets. The proposed framework allowed us to quantify the level of agreement between each ECV temporal change using their own uncertainties. The results are essential for users when ECVs products are assimilated together into a land surface model (Kaminski et al., 2013; Leroux et al., 2018; Wu et al., 2019; Albergel et al., 2020). The sensitivity analysis on the level of confidence of the simultaneous changes allowed us to better understand the relation between the associated uncertainties of each ECV and its impact on the product's agreement and biases. Furthermore, the impact of uncertainties used to aggregate the products into different spatial resolutions was also analysed. This analysis was important, since frequently ECVs are aggregated separately to fit model resolution and physical consistency is impacted (Kaminski et al., 2017).

Our results showed that agreement between the ECVs' changes varied significantly through each dataset. Not only because the agreement differed in terms of magnitude but also temporally and spatially, meaning that for certain regions and periods, products reveal a lack of consistency:

● CGLS products exhibited a variation in the agreement and biases between the SPOT-VEGETATION and PROBA-V periods. This was already observed on version 1 of the product (Mota and Gobron, 2017, Cammalleri et al., 2019). Our findings identified that the agreement between these FAPAR and LAI changes was severely affected by temporal trends. Although some validation efforts showed temporal consistency in the ECV time series (Camacho et al., 2013), our results demonstrated that there was a lack of temporal consistency in associated uncertainties, mainly due to the transition of sensors. As revealed by the non-significant bias trends, the uncertainties magnitudes were changing over time: the LAI uncertainties were getting smaller or the FAPAR uncertainties increased. The agreement dependency with land cover classes showed a lack of spatial consistency. There was a high dependence on seasonality indicating that the products changes were not consistent, mainly during the periods of small/constant vegetation changes.
● The agreement between the LAI and FAPAR from JRC-TIP was always very high. This was primarily because FAPAR was retrieved from the two-stream model after state variables were retrieved. The relative uncertainties of each ECV were also larger compared to other products, as the prior values were higher. Consequently, this led to a low number of change cases available to analyse the agreement sensitivity during the growing and senescence periods. If the priors' values are being restricted to lower range values, the uncertainties will decrease. JRC-TIP was also the only dataset where ECV agreement was less affected by LC, seasonality and trends: this is mainly because the retrieval algorithm is independent of land cover types, as no biome assumption was made.
● The MCD15 agreement consistency was close to the JRC-TIP one, and in some cases statistically identical. On some occasions, it was characterized by higher agreement than for the JRC-TIP in sensitivity to the growth and senescence period. This could be due to the higher temporal resolution (8-day) used here in contrast to that of JRC-TIP (16-days) but also because their reported uncertainties were smaller and therefore contributed more to the agreement metrics. The MCD15 also showed that the sensitivity to decrease was a little higher than for increases. This could reflect the fact that in the senescence period, leaf yellowing and the fall was quicker and easier to monitor. However, in contrast to the other datasets where agreement trends were globally distributed, trends for the MCD15 product were mainly identified over the eastern hemisphere. Furthermore, these products were the ones mostly affected by non-coherence changes between LAI and FAPAR, meaning that significant changes were associated with physically inconsistent situations.

Our results showed that the ECV's uncertainty contribution to the calculation of the level of confidence of the changes was different for each product and land cover class. This suggested that different uncertainty definitions between each product impacts depend on the vegetation type. For the JRC-TIP, uncertainties propagate consistently according to its formulation, *i.e.*, in simplistic terms a negative exponential based on LAI is used to calculate FAPAR and therefore results in lower relative uncertainties for FAPAR than for LAI. The confidence threshold sensitivity analysis confirmed that the uncertainty propagation, in the JRC-TIP case, was in accordance with the physical equation relating the two ECVs. For MCD15, the results showed that there was an inversion in the relative magnitude of the ECV uncertainties: the LAI relative uncertainties tended to be smaller than the FAPAR ones. As they were calculated using the standard deviation of the retrieval solutions, during the retrieval algorithm, the range of values was larger in LAI than for the FAPAR. Exceptions can only be observed for small LAI values. As for CGLS product, agreement between the ECV changes was characterized by the larger LAI uncertainties, with minor expectations for extreme LAI values. The CGLS products uncertainties were unconnected as each ECV was derived directly from independent neural network algorithms. Therefore, there was no uncertainty relation between them.

This study also indicated that spatial resolution had an impact on the level of consistency of the ECV's changes. On average, higher agreement and low biases always resulted from lower resolution resampled products when using weighting averages based on the associated uncertainties. This meant that having a higher number of pixels (more information) leads to a low probability of non-coherence between the ECV's changes. At the same time, aggregation tends to smooth changes and lead to a decrease in sensitivity. However, because uncertainty is used to weigh each gridcell average, products that perform worse, or deviate significantly from the trend, over a particular land cover, could suggest that there is a problem on how uncertainties are estimated and therefore their use needs further research.

In conclusion, JRC-TIP and MCD15 datasets offered a higher agreement between the LAI and FAPAR temporal variations and were spatially and temporally consistent. The MCD15 product results were similar to those of the JRC-TIP, but it was characterized by smaller relative ECV uncertainties that can also indicate a lack of coherence between LAI and FAPAR changes. The CGLS datasets had less consistency, especially over small vegetated cover that was either higher or lower during the growing and senescence periods.

This study proposed a framework to allow identification of physical consistency in the agreement between terrestrial ECV changes with various metrics made available temporally and spatially. Depending on the application, users that aim to assimilate these ECVs into land surface models, namely global vegetation models, need to fully understand where, and when, inconsistencies can occur. This study demonstrated that product retrieval algorithms that are independent performed worse in terms of agreement than those physically based. Furthermore, uncertainty needs to be better defined and evaluated for its fitness for purpose. At this stage, none of these EO products has its uncertainty fully characterized by propagating L1 radiance uncertainties to the L2 biophysical products. This can lead to large differences between the products on what effects are considered in their uncertainties. With the increasing number of Earth Observation satellite programs, validation of the available ECV products and their uncertainties was not only becoming more important in context of product accuracy, but also in terms of physical compatibility. The proposed framework integrated both information on the temporal changes and the associated uncertainties of these products to assess physical consistency before any assimilation studies. This methodology can be applied to any type of ECVs if a physical link is known. The advantage is the economy of computational demands when compared to assimilation methodology. Also, it does not depend on other land variables and model scheme. It also provides information at both spatial and temporal scale for detecting any bias when multiple sensors are used or when assumptions are made in the retrieval algorithm, thus potentially helping producers to refine them. However, a necessary condition is having product uncertainties with the same unit and definition.

## Declaration of Competing Interest

The authors declare that they have no known competing financial interests or personal relationships that could have appeared to influence the work reported in this paper.

## Appendix A Supplementary data

Supplementary material Image 1
