# Supplementary material for: Cross-ECV consistency at global scale: LAI and FAPAR changes
Source: Remote Sens Environ. 2021 Sep 15;263:112561. doi: 10.1016/j.rse.2021.112561 (PMC8299548; doi:10.1016/j.rse.2021.112561)
Supplement: Supplementary file 1 — Supplementary material [file mmc1.docx]

# Supplementary material

## Land cover map

Throughout this study, some of the results are presented by land cover type. The landcover product that was used is detailed in section 2.4. In addition, in order to simplify and allow for a better interpretation of the resulting landcover class differences, the results were also aggregated into types of vegetation structure. For this we adjusted the IPCC class corresponding legend to monitor landcover change, as suggested in the CCI landcover product user guide V2, pag. 30.


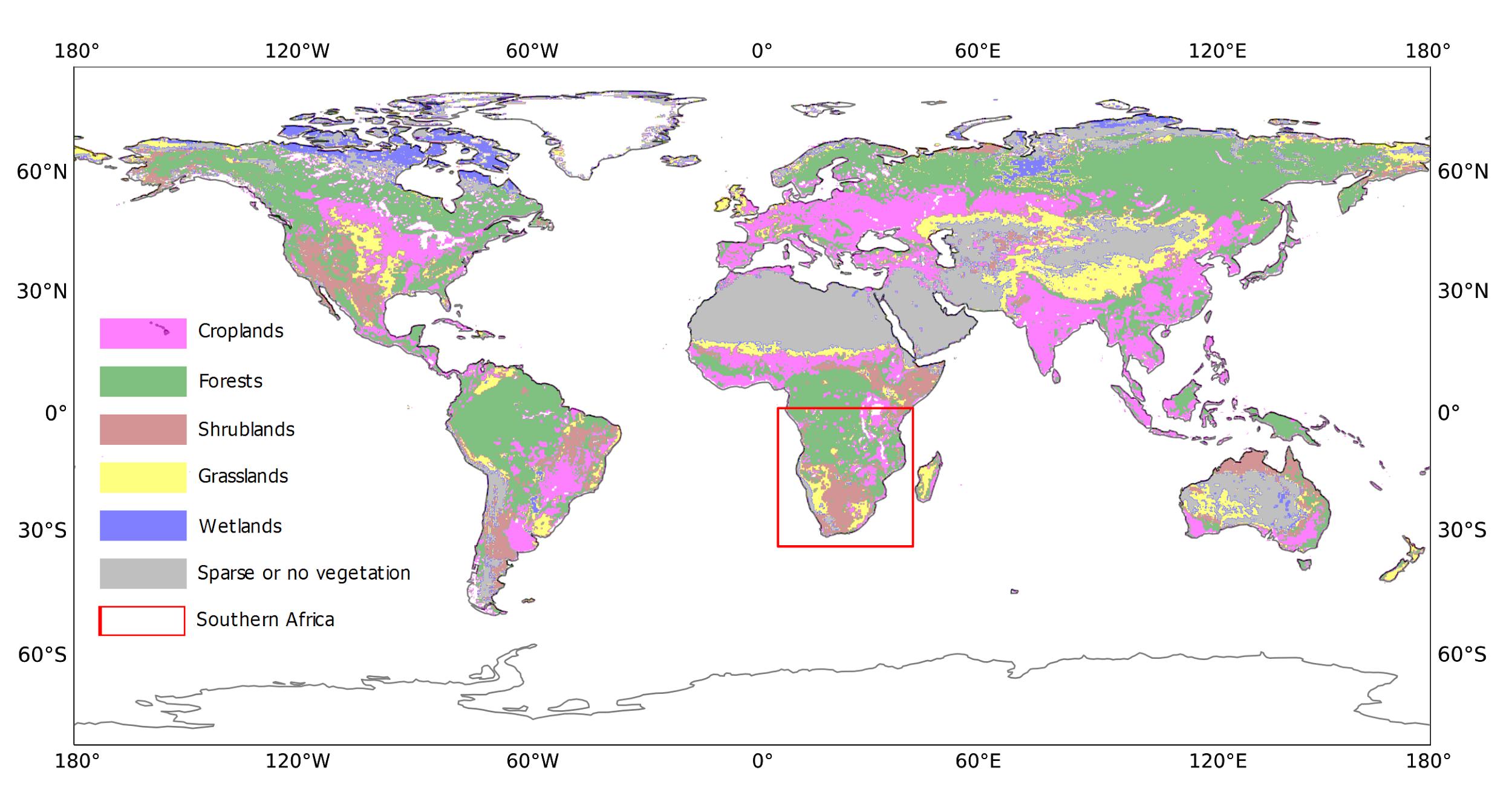


Figure S1: Spatial distribution of the land cover classes aggregated by vegetation structure. The vegetation structure map is based on the CCI landcover product, epoch 2015, and the aggregation class correspondence is outlined in table S1.

Table S1: Climate Change Initiative Land Cover class (CCI-LC) aggregated by vegetation structure adapted from the International Panel for Climate Change (IPCC) class legend for monitoring change detection.

| **Adjusted IPCC Classes** | **LCCS legend for CCI-LC** | |
| --- | --- | --- |
| Croplands | 10 | Rainfed cropland |
|  | 20 | Irrigated cropland |
|  | 30 | Mosaic cropland (>50%) / natural vegetation (tree, shrub, herbaceous cover) (<50%) |
|  | 40 | Mosaic natural vegetation (tree, shrub, herbaceous cover) (>50%) / cropland (< 50% |
| Forests | 50 | Tree cover, broadleaved, evergreen, closed to open (>15%) |
|  | 60, 61 | Tree cover, broadleaved, deciduous, closed to open (> 15%) |
|  | 70, 71 | Tree cover, needleleaved, evergreen, closed to open (> 15%) |
|  | 80 | Tree cover, needleleaved, deciduous, closed to open (> 15%) |
|  | 90 | Tree cover, mixed leaf type (broadleaved and needleleaved) |
|  | 100 | Mosaic tree and shrub (>50%) / herbaceous cover (< 50%) |
| Shrublands | 120 | Shrubland |
| Grasslands | 110 | Mosaic herbaceous cover (>50%) / tree and shrub (<50%) |
|  | 130 | Grassland |
| Wetlands | 160 | Tree cover, flooded, fresh or brakish water |
|  | 170 | Tree cover, flooded, saline water |
|  | 180 | Shrub or herbaceous cover, flooded, fresh-saline or brakish water |
| Sparse or no vegetation | 190 | Urban |
|  | 140 | Lichens and mosses |
|  | 150 | Sparse vegetation (tree, shrub, herbaceous cover) |
|  | 200 | Bare areas |

## Continental agreement metrics.

To complement the results on the spatial consistency of the agreement and bias metrics grouped by land cover class, we show here the corresponding results over the other continents.


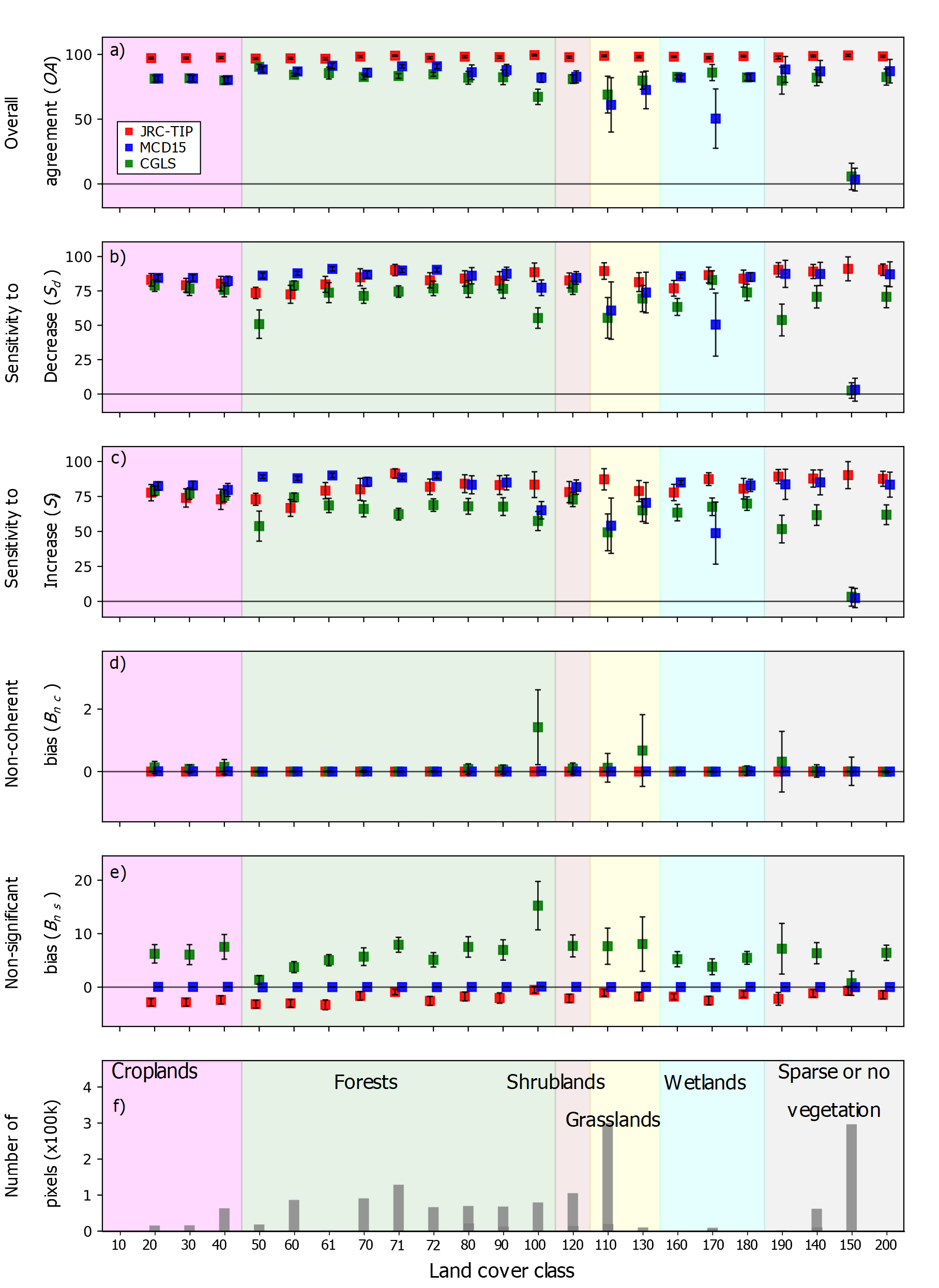


**Figure S1:** Spatial mean agreement metrics between the three LAI and FAPAR datasets by land cover class over the North American continent using the 50% confidence threshold of change (error bar indicates one standard deviation).


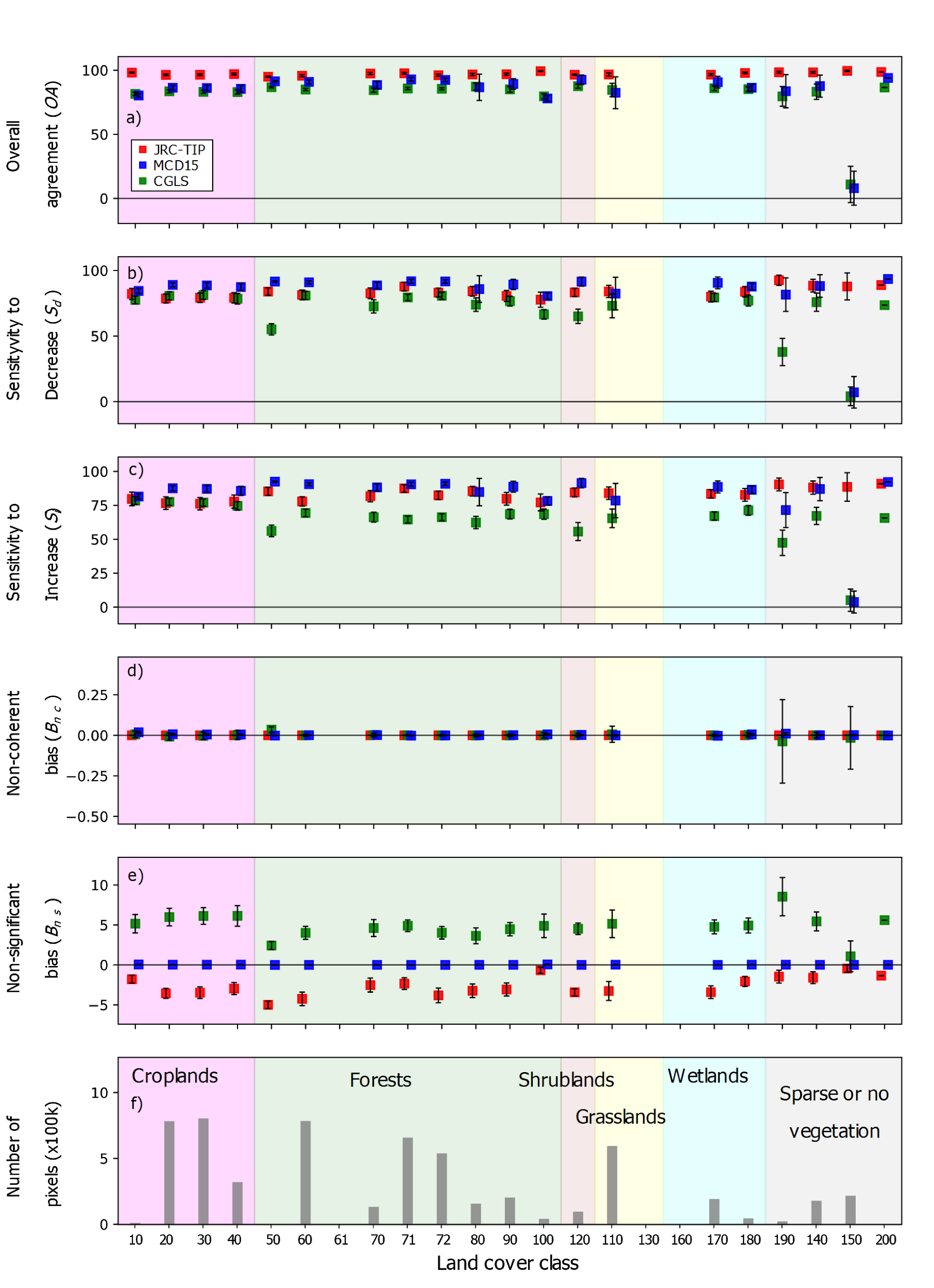


**Figure S2:** Spatial mean agreement metrics between the three LAI and FAPAR datasets by land cover class over the European continent using the 50% confidence threshold of change (error bar indicates one standard deviation).


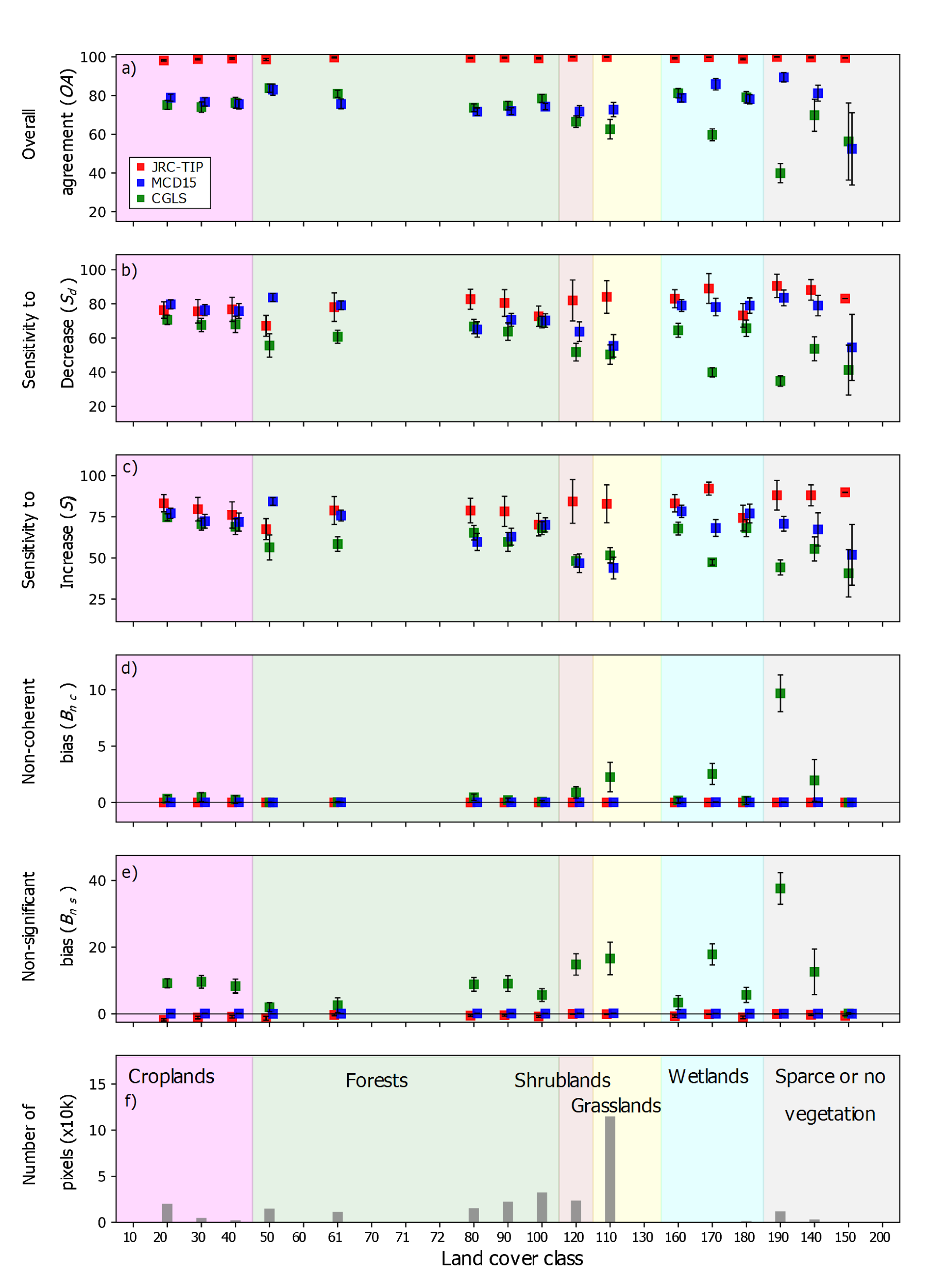


**Figure S3:** Spatial mean agreement metrics between the three LAI and FAPAR datasets by land cover class over the Australian continent using the 50% confidence threshold of change (error bar indicates one standard deviation).


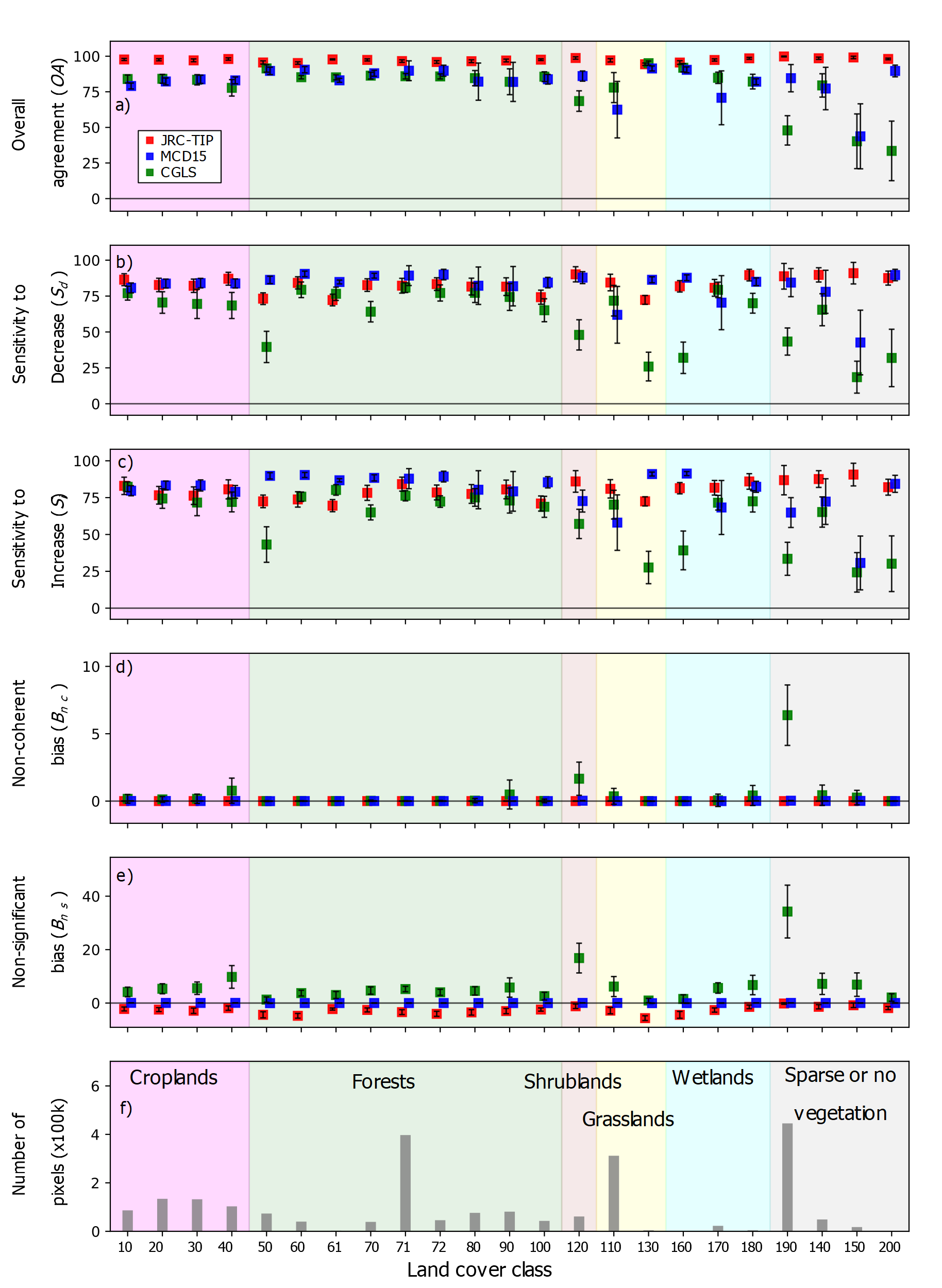


**Figure S4:** Spatial mean agreement metrics between the three LAI and FAPAR datasets by land cover class over the Asian continent using the 50% confidence threshold of change (error bar indicates one standard deviation).


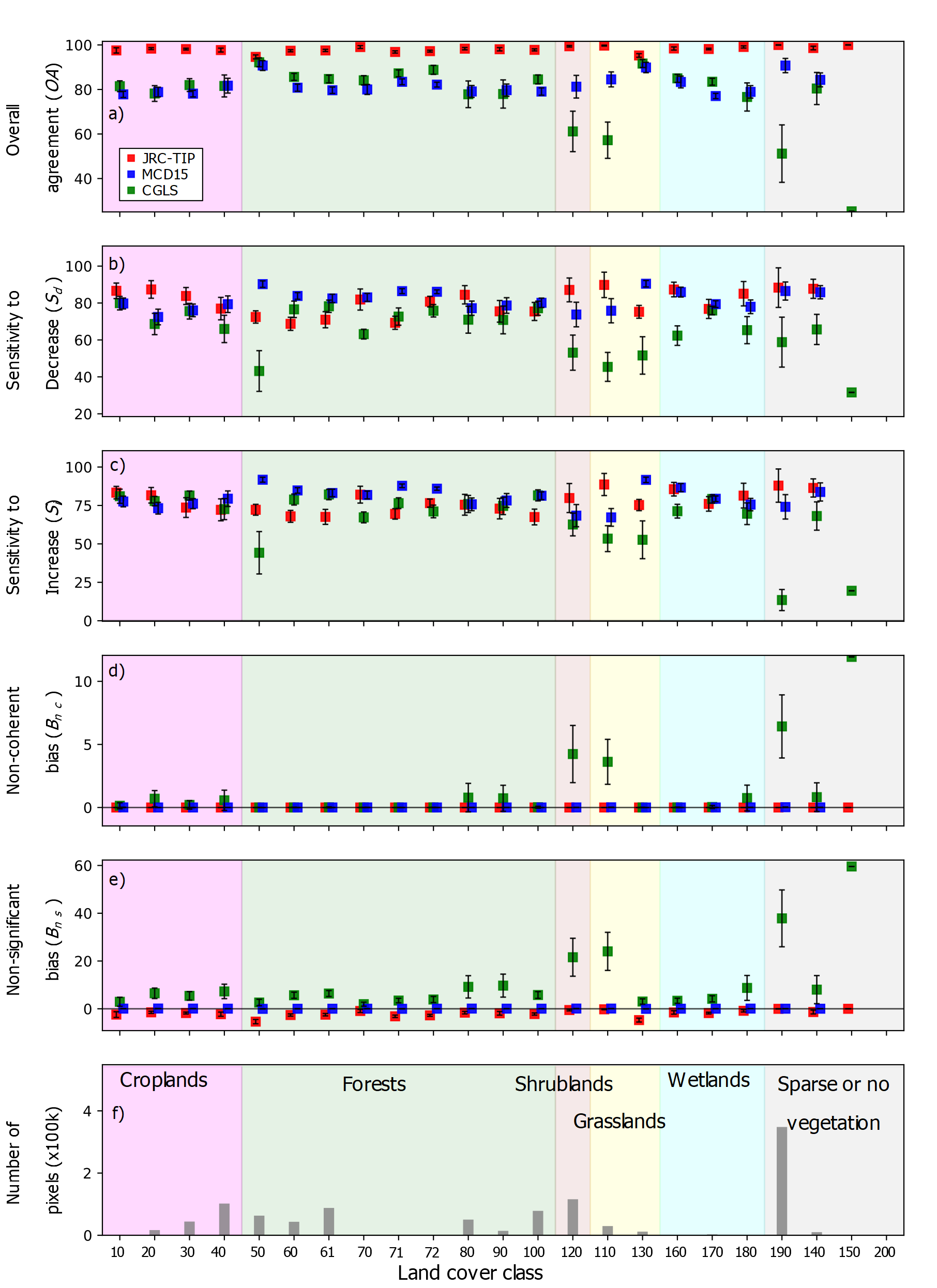


**Figure S5:** Spatial mean agreement metrics between the three LAI and FAPAR datasets by land cover class over the African continent using the 50% confidence threshold of change (error bar indicates one standard deviation).
